# Relationship Between Body Mass Index and Body Fat Percentage in a Group of Indian Participants: A Cross-Sectional Study From a Tertiary Care Hospital

**DOI:** 10.7759/cureus.47817

**Published:** 2023-10-27

**Authors:** Raiza Rai, Tamoghna Ghosh, Sunil Jangra, Shweta Sharma, Sujata Panda, Kanwal P Kochhar

**Affiliations:** 1 Department of Physiology, All India Institute of Medical Sciences, New Delhi, New Delhi, IND; 2 Department of Medicine, All India Institute of Medical Sciences, New Delhi, New Delhi, IND

**Keywords:** overweight and obesity, community obesity, nutrition and metabolism, percent body fat, body mass index (bmi )

## Abstract

Background

Body mass index (BMI) is an important indicator of overweight and obesity. Unlike BMI, body fat percentage (BF%) can be utilized to estimate body composition regardless of weight and height. The association between BMI and BF%, as well as the impact of age and gender, may help estimate the prevalence of obesity more clearly. This study aimed to assess the relationship between BMI and BF%, examine the effect of age and gender on this relationship, and establish the linearity/curvilinearity of this relationship.

Methodology

The body composition analysis of 1,150 participants in various institutional events (institution foundation day) during 2019 and 2023 was performed using the Accuniq bio-electrical impedance analyzer (BIA) (Accuniq, Netherlands). The participants included undergraduate, postgraduate medical, and PhD students, as well as employees of All India Institute of Medical Sciences, New Delhi. Age groups were categorized as under 17 years, young adults (18-25 years), adults (26-44 years), middle-aged adults (45-59 years), and older adults (≥60 years). Pearson’s correlation coefficient (r) was used for analyzing the relationship between BMI and BF%. To assess the effect of age and gender on this relationship, multiple regression analysis was applied, and polynomial regression was applied to test its linearity. The data were analyzed using SPSS version 25 (IBM Corp., Armonk, NY, USA).

Results

Males made up a larger proportion of the participants (56.3%; 647). The mean age of the participants was 36.5 ± 13.6 years. The mean BMI and BF% were 24.7 ± 4.0 kg/m^2^ and 29.1 ± 8.7%, respectively. A significant and moderate positive correlation (r = 0.630, p < 0.01) was observed between BMI and BF%. The mean ± SD of BMI and BF% had a directly proportional relationship with age. Among both genders, females showed a greater correlation (r = 0.852). Both age and gender had a significant effect on this relationship, with gender impacting more than age (β = 0.488, p < 0.000). The curvilinear nature of the relationship between BMI and BF% was demonstrated with the female model showing a more precise fit (R^2^ = 0.72, standard error of the estimate = 3.3%).

Conclusions

The relationship between BMI and BF% was significant and positive in this group of Indians. This relationship was significantly impacted by age and gender and was curvilinear in nature. Females had a higher association than males between BMI and BF%. The study suggests that BMI, BF%, and the effects of age and gender should be taken into consideration when predicting obesity.

## Introduction

The growing epidemic of obesity and overweight has been making inroads upon nutritional and public health as a serious issue and can potentially put a burden on healthcare expenditures [1,2]. The World Health Organization defines overweight and obesity as a kind of malnutrition caused by excessive or abnormal fat accumulation, which puts health at risk. Obesity is a lifestyle-related, non-communicable disease and a risk factor for several other disease conditions such as type 2 diabetes mellitus (T2DM) and heart diseases [3,4]. Obesity-related comorbidities such as hypertension, metabolic syndrome, dyslipidemia, T2DM, cardiovascular disease, non-alcoholic fatty liver disease, obstructive sleep apnea, and certain cancers are becoming more widespread in India [5].

There are about 1.9 billion overweight people worldwide [6], and according to the 2022 Global Nutrition Report, 677.6 million adults are obese, with more women being obese (393.5 million, 15.1%) than men (284.1 million, 11.1%) [7]. Over 2 billion individuals (44%) were overweight or obese in 2016, with more than 70% living in low- or middle-income nations, shattering the misconception that obesity is solely an issue in high-income countries. The fact that 55% of the increase in obesity is occurring in rural areas highlights the severe potential economic and health consequences. Globally, in 2016, the prevalence of overweight/obesity among adults was higher in males (29%) than in women (25%); nevertheless, 19% of women were overweight/obese compared to 15% of men [2]. According to the global burden of disease, each year over 4 million people lose their lives due to being overweight or obese. Between 2010 and 2040, the incidence of overweight is expected to more than double among Indian adults aged 20 to 69, while the prevalence of obesity is expected to triple [8]. According to the National Family Health Survey (NFHS-5, 2019-2021), among Indian adults aged 15 to 49, there has been a consistent drop in being thin and a steady increase in being overweight or obese (from 19% to 23% among males and 21% to 24% among females from NFHS-4 (2015-2016) to NFHS-5) [2,4].

There is still a lack of clarity regarding whether low physical activity or overconsumption of energy-rich foods is principally linked to overweight or obesity. Long-term physical inactivity in the adult population of Copenhagen was not associated with the development of obesity, but it was suggested that obesity may lead to physical inactivity [9]. Physical activity was found to be inversely related to glucose intolerance, obesity, and central fat distribution, particularly in Pima Indian men [10]. Lack of substantial physical activity among 34% of Indians was estimated between 2001 and 2006 by a Lancet study [11]. Global analysis has shown an association between increased consumption of high-calorie foods and increased average body weight of the population, particularly in high-income nations [12]. A study on Pima Indians discovered that total energy intake and resting metabolic rate had a significant positive association with changes in body weight. However, changes in body weight were not related to changes in energy expenditure due to physical activity [13]. Individuals consuming high-fat and high-sugar foods and animal products have been reported to exhibit significantly higher body sizes [14].

Body mass index (BMI) has been a crucial indicator in the classification of obesity. While BMI is a general measure of nutritional adequacy, body fat percentage (BF%) is a better predictor of visceral fat mass and an independent risk factor for cardiovascular disease, diabetes, and metabolic disorders. Weight gain corresponds with an increase in BMI, however, the gain in weight could be attributable to a variety of factors, such as an increase in muscle mass, an increase in adiposity, or an increase in bone density. As BMI does not distinguish between increasing mass in the form of fat, lean tissue, or bone, it may not be sufficient to evaluate the health risks associated with increased adiposity. As a result, considerable misclassification occurs [8,15]. The fact that BMI only considers height and weight and not the distribution and percentage of BF makes it even more imperative to combine the measurement of BMI and BF% to acquire a better picture of the body adiposity and prevalence of obesity. Bioelectrical impedance analysis (BIA) is a technique that is widely utilized in body composition research due to its fast information processing, non-invasiveness, and rapid information output [16]. This study aimed to examine the relationship between BMI and BF% of Indian participants by collecting body composition data using BIA.

## Materials and methods

Study settings

A cross-sectional analytical study was conducted among volunteers/attendees aged 17-84 years during 2019-2023 (excluding 2020 due to the COVID-19 lockdown). The convenience sampling approach was used, and participants were chosen from a specific group of attendees at institutional events (institution foundation day). During this time, the participants attended a total of four (one per year) institution foundation days hosted by the All India Institute of Medical Sciences (AIIMS), New Delhi, and their written consent was obtained.

Sample size

Assuming a correlation of 0.1 based on the study by Ranasinghe et al. (2013) [17], at 80% power, and 5% level of significance, the sample size was calculated at 761. Therefore, we decided to recruit 1,150 participants in this study.

Inclusion criterion

All MBBS and nursing students in undergraduate (UG) and postgraduate (PG) courses, PhD students, and employees of AIIMS, New Delhi who were willing to volunteer and undergo BIA were included.

Exclusion criterion

Individuals who were not willing to participate and undergo BIA during the institutional events were excluded.

Study methodology

Age groups were classified as under 17 years, young adults (18-25 years), adults (26-44 years), middle-aged adults (45-59 years), and old age (≥60 years). Height was measured without footwear in a standing position. The BIA from Accuniq (Netherlands) was used to estimate BF% and body weight with minimum clothes and no accessories. The method of measurement used was a tetra-polar electrode method using eight touch electrodes. The equipment measured whole body impedance from the hands to the feet by applying an electric alternating current flux of 180 A 15 at frequencies of 5, 50, and 250 kHz [18].

Ethical considerations

Ethical approval was obtained from the Institute Ethics Committee, AIIMS, New Delhi (approval number: IEC-314/03.05.2019).

Data analysis

Pearson’s correlation coefficient (r) was determined to examine the relationship and degree of relationship between BMI and BF% regarding gender and age variables. Descriptive statistics such as mean, standard deviation (SD), and percentages were reported. Multiple regression analysis for estimating the effect of age and gender on the BMI and BF% relationship was applied. The linearity of the BMI and BF% relationship was investigated using polynomial regression analysis. Statistical analysis was performed using SPSS version 25 (IBM Corp., Armonk, NY, USA).

## Results

Relationship between BMI and BF%

**Figure 1:**
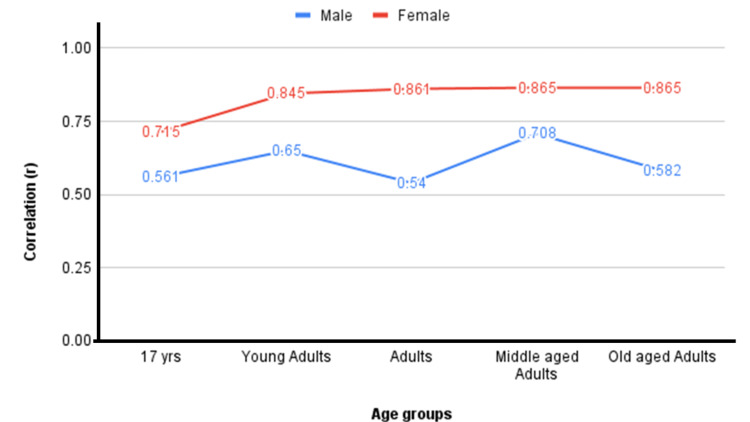
Graphical representation of the comparison of the correlation between BMI and BF% across age groups in males and females. BMI = body mass index; BF% = body fat percentage

In this study, 1,150 participants underwent BIA, with 56.3% (n = 647) being males. The BMI values in the study sample ranged from 14.4 to 37.6 kg/m^2^. A total of 11.13% of our study sample had BMI >30 kg/m^2^. As shown in Table 1, across all age groups, old-aged males and females had the highest BMI and BF%. The correlation between BMI and BF% was calculated to be significant and moderately positive (r = 0.630, p < 0.01). A significant and weak positive correlation was observed between age and BMI (r = 0.271, p < 0.01) and age and BF% (r = 0.241, p < 0.01). BMI and BF% correlated strongly in females (r = 0.852, p < 0.01) and moderately in males (r = 0.626, p < 0.01). For all age groups, the correlation was estimated to be significant and positive (p < 0.01) with moderate correlation in age 17 (r = 0.524), young adults (r = 0.606), adults (r = 0.584), and old-aged adults (r = 0.613) and strong correlation in middle-age adults (r = 0.701). The correlation across all age groups in males and females is shown in Table 2 and Figure 1. Correlation based on occupation was significant and moderate in undergraduate students (r = 0.575), PhD students (r = 0.659), staff/employees (r = 0.603), and retired staff (r = 0.628) and significant and strong in postgraduate students (r = 0.793).

**Table 1:** Mean ± standard deviation values for age, BMI, and BF% in males and females across age groups. BMI = body mass index; BF% = body fat percentage

Variable	Female (n = 503)					Male (n = 647)				
	17 year olds	Young adults	Adults	Middle-aged adults	Old-aged adults	17 year olds	Young adults	Adults	Middle-aged adults	Old-aged adults
BMI (kg/m^2^)	22.2 ± 3.9	22.7 ± 4.3	25.2 ± 4.1	26.4 ± 3.5	26.4 ± 4.3	21.7 ± 3.7	23.0 ± 4.1	24.7 ± 3.6	25.2 ± 3.1	26.5 ± 4.5
BF%	34.3 ± 10.2	31.8 ± 7.4	34.5 ± 5.9	36.0 ± 5.6	37.6 ± 5.7	20.5 ± 7.8	22.1 ± 8.8	24.9 ± 7.8	26.2 ± 6.0	31.5 ± 7.1
Age (years)	17.2 ± 0.6	21.2 ± 2.5	33.9 ± 5.6	51.1 ± 4.3	66.5 ± 7.7	17 ± 0.0	20.8 ± 2.7	33.8 ± 4.7	50.8 ± 3.5	69.9 ± 6.0
N	9	80	284	98	32	19	148	297	148	35

**Table 2:** Correlation between BMI and BF% in males and females across age groups. BMI = body mass index; BF% = body fat percentage

Gender	17 year olds	Young adults	Adults	Middle-aged adults	Old-aged adults
Male	0.561	0.650	0.540	0.708	0.582
Female	0.715	0.845	0.861	0.865	0.865

Effect of age and gender on the BMI and BF% relationship

Multiple regression showed that gender had a greater effect on the BMI and BF% relationship than age (Table 3). BMI contributed more as a significant predictor variable in females (β = 0.869, p < 0.000) than in males (β = 0.484, p < 0.000), as shown in Table 3 and Table 4.

**Table 3:** Multiple regression analysis to determine how BMI, age, and gender affect changes in BF%. BMI = body mass index; BF% = body fat percentage

	Intercept/Regression coefficients/R^2^	SE	Beta
Intercept	-12.726	1.074	
BMI	1.065	0.039	0.532 (p < 0.000)
Age	0.060	0.013	0.088 (p < 0.000)
Gender	9.149	0.354	0.488 (p < 0.000)

**Table 4:** Multiple regression analysis to determine how the BF% changes with BMI and age for both males and females. BMI = body mass index; BF% = body fat percentage

	Male				Female		
	Intercept/Regression coefficients/R^2^	SE	Beta	Intercept/Regression coefficients/R^2^		SE	Beta
Intercept	1.835	1.655		4.067		0.812	
BMI	0.943	0.065	0.484 (p < 0.000)	1.194		0.030	0.869 (p < 0.000)
Age	0.094	0.021	0.153 (p < 0.000)	0.013		0.011	0.000 (p < 0.000)
R^2^	0.289			0.764			

Linearity/curvilinearity between BMI and BF%

**Figure 2:**
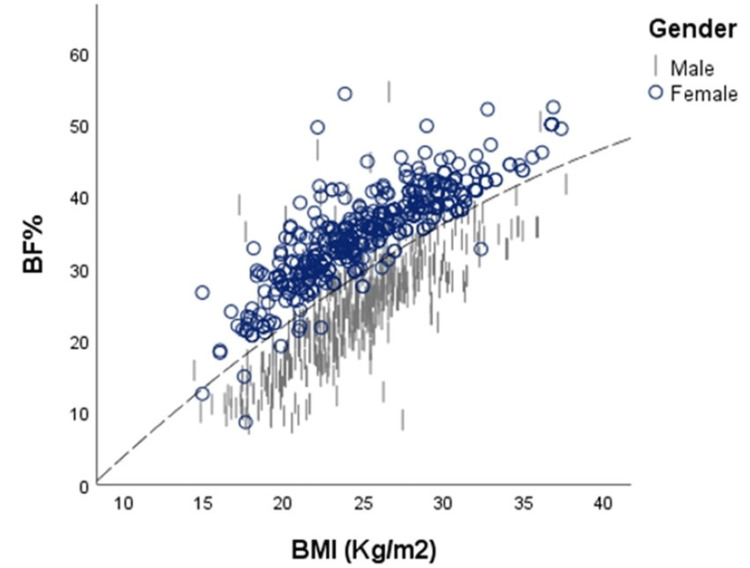
Grouped scatter plot representing the relationship between BMI and BF% in males and females. BMI = body mass index; BF% = body fat percentage

In the linear model, for males, 39.2% of the variation in BF% was accounted for by BMI, and for females was estimated to be 72.5%. On adding the quadratic component, the variance increased by 0.4% (p = 0.043) in males and by 15% (p < 0.000) in females. In comparison to the male model (R^2^ = 0.39, standard error of the estimate (SEE) = 4.1%), the female model (R^2^ = 0.72, SEE 3.3%) offered a more precise fit. The curvilinear nature of the association between BMI and BF% is demonstrated in Figure 2.

## Discussion

We aimed to determine the relationship between BMI and BF% in this group of Indians aged 17-84 years, the influence of age and gender on this relationship, as well as its linearity/curvilinearity. Several studies have investigated the relationship between BMI and BF% across diverse Asian population groups [17,19-23]. Some have also investigated the predictive influence of race in the BMI and BF% relationship [24-29]. We found that the mean BF% was higher in females than in males across all age strata. This could be explained by understanding the physiological and hormonal variations between the sexes. Biologically, women have higher levels of fat, which is necessary for reproductive functions and hormone regulation. Fat is stored in various parts of the body, such as breasts, pelvis, and thighs. These higher levels of fat contribute to the higher mean BF% in females. Conversely, males typically have a higher percentage of lean body mass, which includes muscle and bone mass. Testosterone, the primary male sex hormone, plays a role in promoting muscle development and may lead to higher muscle mass in men compared to women [30].

Our findings of this Indian group confirmed a significant positive association between BMI and BF%. This relationship was the strongest in middle-aged adults. Females in all age groups demonstrated a strong and significant positive correlation between BMI and BF%, while only middle-aged adult males had a strong correlation for this relationship. In females, the strength of this association increased with age compared to males. In both genders, with advancement in age, the mean ± SD of BMI as well as BF% showed a steady increment. The multiple regression analysis indicated that age and gender had a significant impact on the relationship between BMI and BF%, with gender having a larger impact (β = 0.488, p< 0.000). This effect of gender is in line with previous studies [24,25,29]. The polynomial regression determined the relationship between BMI and BF% to be curvilinear in nature, with females (p < 0.000) having a better fit to the curve than males (p = 0.043). A growing number of studies suggest that the link between BMI and BF% varies greatly across populations, and there is a divergence in conclusions regarding whether this association is linear or curvilinear. A cross-sectional study among Sri Lankan adults estimated a strong positive curvilinear correlation in males (r = 0.75) and females (r = 0.82). This curvilinear effect was stronger in females than in males [18]. Between white and black adults in the United States, a quadratic relationship demonstrated that the association is neither age- nor gender-independent [24].

With the help of body composition analysis (BCA), we can predict the health of healthcare workers and students during their jobs, studies, and even after retirement, which gets affected due to their stressful work schedule. This study also elicits whether BCA can be used as a marker for their health, especially for lifestyle diseases such as obesity, overweight, and visceral fat in addition to subcutaneous fat. The novelty of this study was to elicit health literacy and nutrition awareness about BF%, BMI, and the effect of age and gender on these health parameters among healthcare workers (laboratory technical officers) and medical postgraduate and undergraduate students who interact with patients and caregivers. By addressing the issue of obesity among healthcare workers and students, healthcare institutions can promote a culture of preventive healthcare. Educating and supporting the workforce and students to maintain a healthy lifestyle can not only help reduce healthcare costs but also set an example for patients and the broader community.

Our study had several limitations. First, we cannot generalize these findings to all Indians because the sample was drawn from a health-conscious group of Indians who attended medical institutional events. Second, bioelectrical impedance is not often regarded as an excellent reference as traditional body composition techniques such as hydrodensitometry or water dilution techniques as well as multicomponent models based on measurements acquired from multiple reference body composition techniques. Third, there was a lack of gender balance (the number of males and females was not equal), with males being higher in proportion than females, which may have affected the difference in the influence of males and females as well as the influence of gender on the BMI and BF% association. Fourth, as our study concentrated on the association between BMI and BF% and the impact of age and gender on it, we did not take into account different confounding variables that may affect body composition, such as physical activity, dietary intake, and smoking status. Finally, as this was a cross-sectional and not a longitudinal study, we could not discover any changes in the BMI and BF% over a period of time due to any confounding variables mentioned above or due to the effect of any intervention. Further studies on this topic may consider these factors for better results.

## Conclusions

The results of our study demonstrate a moderate positive relationship between BMI and BF% in this group of Indians, as estimated by BIA. The curvilinear nature of the BMI and BF% relationship was also confirmed, and both age and gender significantly affected this relationship, with gender having a more pronounced effect. According to the study, when estimating obesity in people, BMI, BF%, as well as the impact of age and gender, should be taken into account.
